# Simvastatin-Encapsulated Topical Liposomal Gel for Augmented Wound Healing: Optimization Using the Box-Behnken Model, Evaluations, and In Vivo Studies

**DOI:** 10.3390/ph17060697

**Published:** 2024-05-28

**Authors:** Mohamed Rahamathulla, Rahul Pokale, Yousef Al-ebini, Riyaz Ali M. Osmani, Kamal Y. Thajudeen, Ravi Gundawar, Mohammed Muqtader Ahmed, Syeda Ayesha Farhana, Thippeswamy Boreddy Shivanandappa

**Affiliations:** 1 Department of Pharmaceutics, College of Pharmacy, King Khalid University, Abha 61421, Saudi Arabia; rahapharm@gmail.com; 2 Department of Pharmaceutics, JSS College of Pharmacy, JSS Academy of Higher Education and Research, Mysuru 570015, Karnataka, India; rahulpokale0@gmail.com; 3 Department of Cosmetic Science, Faculty of Allied Medical Sciences, Al-Ahliyya Amman University, Amman 19328, Jordan; y.alebini@ammanu.edu.jo; 4 Faculty of Dentistry, Al-Ahliyya Amman University, Amman 19328, Jordan; 5 Department of Pharmacognosy, College of Pharmacy, King Khalid University, Abha 61441, Saudi Arabia; kthajudeen@kku.edu.sa; 6 Department of Pharmaceutical Quality Assurance, Manipal College of Pharmaceutical Sciences, Manipal Academy of Higher Education, Manipal 576104, Karnataka, India; gundawar.ravi@manipal.edu; 7 Department of Pharmaceutics, College of Pharmacy, Prince Sattam Bin Abdulaziz University, Al-Kharj 11942, Saudi Arabia; muqtadernano@gmail.com; 8 Department of Pharmaceutics, College of Pharmacy, Qassim University, Buraidah 51452, Saudi Arabia; a.farhana@qu.edu.sa; 9 Department of Biomedical Science, College of Pharmacy, Shaqra University, Al-Dawadmi Campus, Al-Dawadmi 11961, Saudi Arabia

**Keywords:** liposomes, drug delivery, supramolecular nanocarriers, SIM, Box-Behnken design, wound healing, topical drug delivery, gels

## Abstract

Statins function beyond regulating cholesterol and, when administered systemically, can promote wound healing. However, studies have yet to explore the topical use of statins for wound healing. The present study demonstrated the topical administration of SIM and aimed to formulate, evaluate, and optimize Simvastatin (SIM)-encapsulated liposome gel carrier systems to facilitate successful topical wound healing. Liposomes containing SIM were formulated and optimized via a response surface methodology (RSM) using the thin-film hydration method. The effects of formulation variables, including the 1,2-dioleoyloxy-3-trimethylammoniumpropan (DOTAP) concentration, Span 80 concentration, and cholesterol concentration, on zeta potential (mV), entrapment efficacy (%), and particle size (nm) were studied. The optimized liposome formulation (F-07) exhibited a zeta potential value of 16.56 ± 2.51 mV, revealing robust stability and a high SIM encapsulation efficiency of 95.6 ± 4.2%, whereas its particle size of 190.3 ± 3.3 nm confirmed its stability and structural integrity. The optimized liposome gel demonstrated pseudoplastic flow behavior. This property is advantageous in topical drug delivery systems because of its ease of application, improved spreadability, and enhanced penetration, demonstrating prolonged SIM release. The assessment of the wound healing efficacy of the optimized liposomal gel formulation demonstrated a substantial decrease in wound size in mice on the sixteenth day post-wounding. These findings suggest that the use of liposomal gels is a potential drug delivery strategy for incorporating SIM, thereby augmenting its effectiveness in promoting wound healing.

## 1. Introduction

The process of wound healing is recognized as the most complex health condition to address in patients [1], as it is an intricate, dynamic process that involves an order of controlled events, including inflammation, tissue formation, reperfusion, and tissue remodeling [2], and any interruption in this process may result in a chronic wound. The wound-healing cascade is disrupted in severe clinical diseases and is typically associated with persistent inflammation and oxidative stress, increasing necrosis and tissue loss instead of healing [3]. Wounds are caused by a perturbation of cellular integrity within the standard anatomical framework of the skin, precipitated by factors such as thermal influences (e.g., burns), physiological aberrations (including malignancies and diabetes), or mechanical trauma (as encountered in surgical procedures) [4]. Wounds are vulnerable to microbial contamination, which causes tissue damage and inflammation, interfering with healing. These wound-healing obstacles can lead to severe consequences that significantly reduce the patient’s standard of living. As a result, discovering more effective methods to promote wound healing is essential [5].

Statins competitively reduce cholesterol production via 3-hydroxy-3-methylglutaryl coenzyme A (HMG-CoA) reductase [6]. Statins have recently exhibited a variety of pleiotropic effects beyond their cholesterol-lowering effect. As a result, they have since been recognized as an innovative therapeutic method for an array of clinical conditions [7]. These compounds have antioxidative, anti-inflammatory, immunomodulatory, and antibacterial properties [8]. Topical statin application to a wound could promote microvascular function, lymph angiogenesis, and angiogenesis. They can also serve as immunomodulators and reduce oxidative stress. Consequently, statins can potentially expedite and augment wound healing [9]. SIM, a prominent statin, has recently emerged as a therapeutic agent that enhances wound healing [5]. SIM also has antibacterial effects on Gram-positive bacterial strains, rendering it a viable alternative to numerous routinely used antibiotics [10]. However, SIM has relatively low oral bioavailability (approximately 5%) because of its poor water solubility and substantial first-pass hepatic degradation [11]. Oral SIM administration may trigger a variety of adverse effects, including myopathy and liver complications. As a result, topical SIM delivery can improve drug availability in the region of wounds while reducing the risk of potential adverse effects [12].

Liposomes are thermodynamically stable vesicles composed of multiple symmetrical lipid bilayers separated into two compartments: an aqueous core and a lipophilic region within the lipid bilayer. Hydrophilic drugs can be embedded in the inner aqueous volume, whereas hydrophobic compounds can become entrapped in lipid bilayers [13]. The lipid bilayer structure of liposomes is the most suitable alternative for traditional drug administration methods because of its safety profile [14]. Liposomes have been investigated and revealed to be valuable and convenient carriers for administering topical drugs for various dermatological conditions due to their ability to fulfill multiple roles in adhering to topical application [15]. The lipid–lipid bilayer of liposomes can combine with comparable structures, such as cellular membranes, enhancing skin penetration and distribution efficiency. Liposomes have been widely used in the pharmaceutical and cosmetic industries to deliver bioactive agents. Among topical drug delivery systems, gels have emerged as the most commonly accepted and are characterized by an interconnected polymeric matrix moistened with water and loaded with the drug or drug carriers [16].

The primary aim of this study was to formulate a gel incorporating SIM-loaded liposomes to achieve effective topical wound healing. The optimization of liposome gels is a critical process in developing suitable formulations for novel vesicular platforms. In association with the Box-Behnken design, the response surface method (RSM) was employed to optimize novel vesicular formulations. The variables considered were the DOTAP concentration (X_1_), Span 80 concentration (X_2_), and cholesterol concentration (X_3_); the response variables were the zeta potential (Y_1_), entrapment efficacy (Y_2_), and particle size (Y_3_). Preliminary studies were conducted to identify the levels of these variables. Moreover, the viscosity, rheological properties, and in vitro drug release were examined using the Higuchi, Korsmeyer–Peppas, zero-order, and first-order kinetics models, and the in vivo efficacy of the optimized formulation was assessed using a full-thickness wound model, accompanied by histopathological studies to analyze tissue changes and pathology.

## 2. Results and Discussion

### 2.1. Box-Behnken Design

Using a three-factor, three-level Box-Behnken statistical design, 15 experimental runs were generated, incorporating three center points. The results are given in Table 1, which shows the ranges of Y_1_, Y_2_, and Y_3_ across all formulations as −4.342 ± 1.4 to +16.56 ± 2.5 mV, 80.32 ± 4.9 to 96.32 ± 2.4%, and 140.9 ± 3.5 to 376.3 ± 7.8 nm, respectively. Subsequently, quadratic models were fitted to the observed responses for the 15 formulations, with the linear model emerging as the most accurate. The equations include statistically significant coefficients (*p* < 0.05); within the model framework, a negative coefficient signifies an inverse correlation between the factor and the response variable, while a positive coefficient indicates a positive influence favoring the optimization of the system.

**Figure 1:**
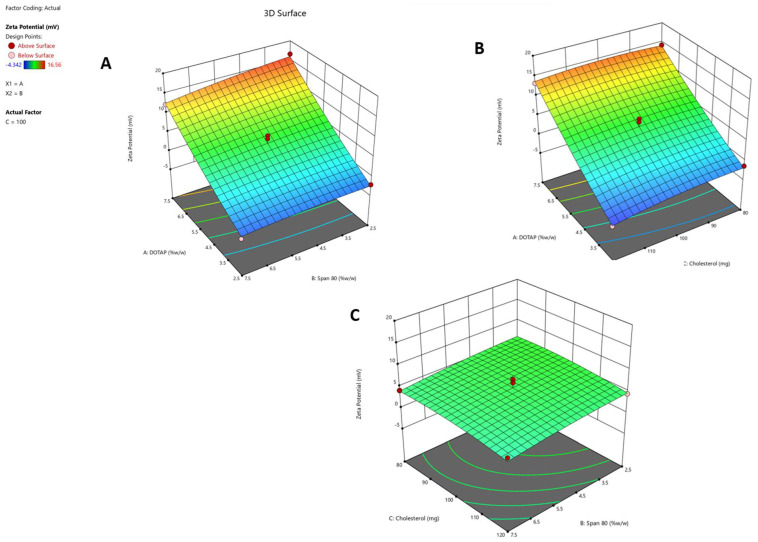
Three-dimensional contour graphs that depict the zeta potential of the Y_1_ response influenced by different independent variables. (**A**) Impact of the X_1_ and X_2_ variables on the Y_1_ response. (**B**) Impact of the X_1_ and X_3_ variables on the Y_1_ response. (**C**) Correlation between the X_2_ and X_3_ variables and their influence on the Y_1_ response’s zeta potential. These contour plots visually represent the relationship between changes in independent variables and variations in the zeta potential of the Y_1_ response. Each experiment was performed in triplicate (*n* = 3).

**Figure 2:**
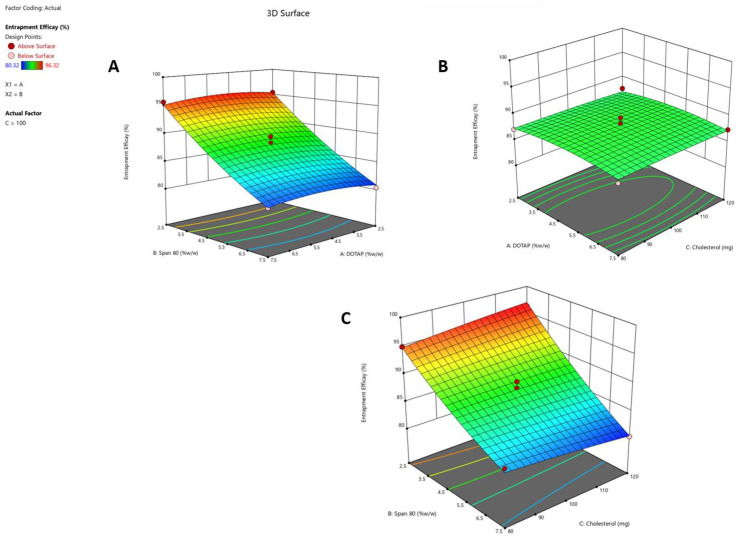
Three-dimensional contour graphs that depict the entrapment efficacy of the Y_2_ response influenced by different independent variables. (**A**) The impact of the X_1_ and X_2_ variables on the Y_2_ response. (**B**) The impact of the X_1_ and X_3_ variables on the Y_2_ response. (**C**) The correlation between the X_2_ and X_3_ variables and their influence on the Y_2_ response’s entrapment efficacy. These contour plots visually represent the relationship between changes in independent variables and variations in the entrapment efficacy of the Y_2_ response. Each experiment was performed in triplicate (*n* = 3).

**Figure 3:**
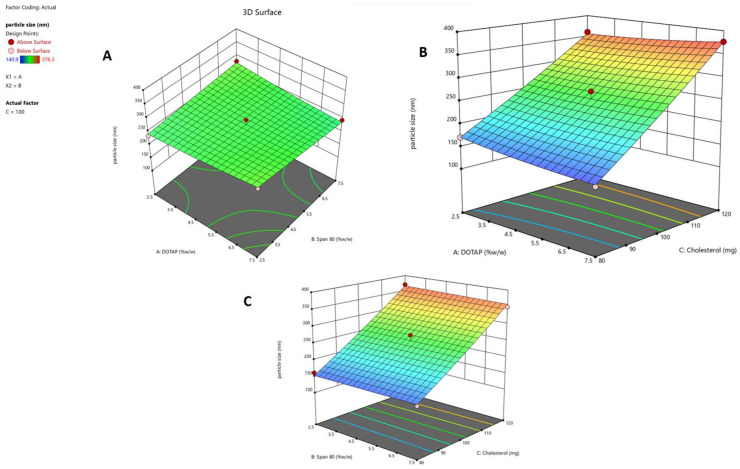
Three-dimensional contour graphs that depict the influence of different independent variables on the particle size of the Y_3_ response: (**A**) the impact of the X_1_ and X_2_ variables on the Y_3_ response, (**B**) the impact of the X_1_ and X_3_ variables on the Y_3_ response, and (**C**) the correlation between the X_2_ and X_3_ variables and their influence on the Y_3_ response’s particle size. These contour plots visually represent the relationship between changes in independent variables and variations in the particle size of the Y_2_ response. Each experiment was performed in triplicate (*n* = 3).

The interactive effects of three independent variables—DOTAP concentration (X_1_), Span 80 concentration (X_2_), and cholesterol concentration (X_3_)—on the responses (Y_1_, Y_2_, and Y_3_) were systematically explored. The response surface was analyzed using contour plots (Figure 1A–C, Figure 2A–C, and Figure 3A–C), corresponding to responses Y_1_, Y_2_, and Y_3_, respectively. These 3D contour plots are well established for examining interactions between variables and responses, providing valuable insights into the simultaneous impact of two factors on responses.

#### 2.1.1. Polynomial Equations Were Used to Analyze the Responses

##### Response 1 (Y_1_): Impact on Zeta Potential

The model formulated the following polynomial equation to describe the variation in the zeta potential:

Y_1_ = +5.3683 + 8.47 X_1_ − 0.97 X_2_ − 0.66 X_3_ − 0.70 X_1_ X_2_ + 0.475 X_1_ X_3_ + 0.03 X_2_ X_3_ 0.72 X_1_^2^ − 0.32 X_2_^2^ − 0.66 X_3_^2^

Y_1_ is the zeta potential, X_1_ is the DOTAP concentration, X_2_ is the Span 80 concentration, and X_3_ is the cholesterol concentration. The model is significant (*p* < 0.001), according to the model’s F value of 18.56. The lack of fit is not substantial, according to the lack of fit F value of 2.80 (*p* = 0.9372). In this instance, X_1_ (the concentration of DOTAP) is a considerable model term and considerably impacts the zeta potential compared to the other parameters. The corrected R^2^ of 0.9186 and the projected R^2^ of 0.9709 agree reasonably well. The precision is adequate and within the desired limit. An acceptable signal is indicated by a ratio of 13.0780. This model can, therefore, be used to explore the design space. The contour graphs in Figure 1A–C illustrate several independent variables affecting the zeta potential (Y_1_). With increasing concentrations of DOTAP, the zeta potential increased significantly. Due to its cationic nature, DOTAP is likely to confer a positive charge to liposomes [17]. To ensure significant particle repulsion and prevent aggregation, colloidal nanosystems are expected to exhibit a zeta potential above −30 mV or below +30 mV [18]. Preliminary screening indicated that elevated concentrations of DOTAP were required to attain a zeta potential exceeding +20 mV. Consequently, a target desirability was set to achieve the desired formulation while maintaining an acceptable level of DOTAP, specifying that the zeta potential should fall within the range of 10–20 mV.

##### Response 2 (Y_2_): Effect on Entrapment Efficacy

The following is a polynomial equation suggested by the model for entrapment efficacy:

Y_2_ = +88.06 + 0.024 X_1_ − 7.09 X_2_ − 0.075 X_3_ + 0.18 X_1_ X_2_ +0.059 X_1_ X_3_ − 1.185 X_2_ X_3_ − 0.78 X_1_^2^ + 0.8725 X_2_^2^ − 0.12 X_3_^2^,

where Y_2_ is the entrapment efficacy. The model was statistically significant (F = 30.41; *p* < 0.001). Compared to the pure error, the lack of fit was insignificant (F = 0.3188; *p* = 0.8160). In this instance, X_2_ and X_2_^2^ are major model variables (i.e., Span 80) and had a greater effect on entrapment efficacy than any other parameter. A negative coefficient indicates that the component has an inverse effect on the response. The projected R-squared and adjusted R-squared are relatively comparable (0.9821 and 0.9498, respectively). The signal-to-noise ratio was measured with adequate precision. The contour plots in Figure 2A–C indicate the effect of several independent variables on entrapment efficacy. According to the results, the EE decreased as the Span 80 concentration increased by more than 5%, which is in agreement with previous research reports [19]. Higher Span 80 concentrations tend to reduce the size of the vesicles, which results in less drug being entrapped.

##### Response 3 (Y_3_): Effect on Particle Size

The following is a suggested polynomial equation based on the model for particle size:

Y_3_ = +253.7 − 1.77 X_1_ − 1.38 X_2_ + 104.38 X_3_ − 17.625 X_1_ X_2_ + 11.275 X_1_ X_3_ − 2.5 X_2_ X_3_ + 4 X_1_^2^ − 0.024 X_2_^2^ + 4.275 X_3_^2^

where Y_3_ is the particle size of the liposomes.

Among the independent factors, X_3_ (i.e., cholesterol concentration) emerged as a significant model term, displaying a positive effect on the particle size of the liposome, as indicated by its markedly high positive coefficient. A positive coefficient for cholesterol implies an increase in particle size at higher concentrations. The overall model is statistically significant (F = 65.05; *p* < 0.0001), and the lack of fit is not significant (F = 0.6063; *p* = 0.6713). The predicted (0.9522) and adjusted (0.9763) R^2^ values are in close agreement. The signal-to-noise ratio was deemed appropriate, with an adequate precision ratio of 22.96, surpassing the threshold of 4. The impact of various independent parameters on particle size is illustrated in the 3D contour plots presented in Figure 3A–C.

### 2.2. Optimization of Formulation Composition

The formulation aimed for response desirability with critical parameters, including entrapment efficiency (≥85%), particle size (≤200 nm), and zeta potential (≥10 mV). The selected composition was selected based on achieving specific values of entrapment efficiency (≥85%), particle size (≤200 nm), and zeta potential (≥10 mV) as desired goals. According to the Box-Behnken design, the following conditions for preparing SIM liposomes were recommended: a 7.36 mg DOTAP concentration, a 2.36% Span 80 concentration, and a 99.78 mg cholesterol concentration, aligning with the specified %EE, particle size, and zeta potential goals. The predicted values for this optimized formulation were a %EE of 93.25%, a particle size of 187.75 nm, and a zeta potential of 15.86 mV, as detailed in Table 1. Following these optimized conditions, the liposomes were prepared, and the actual experimental values for the optimized liposomes were a %EE of 95.6 ± 4.2%, a particle size of 190.3 ± 3.3 nm, and a zeta potential of 16.56 ± 2.5 mV.

### 2.3. Determination of the Entrapment Efficiency (EE%)

The encapsulation efficiency (EE%) values across all formulations ranged from 80.34 ± 0.05 to 96.32 ± 0.04%. Comparable EE% values have been reported in prior studies involving SIM-loaded liposomal vesicles, suggesting a correlation with the intrinsic hydrophobic properties of the drug [19]. Increased concentrations of Span 80 increased the binding affinity of its molecular entities with the bilayers, resulting in pore development [20]. For the optimized formulation, F-07, the EE% was determined to be 95.6 ± 0.02.

### 2.4. Zeta Potential and Vesicular Size

**Figure 4:**
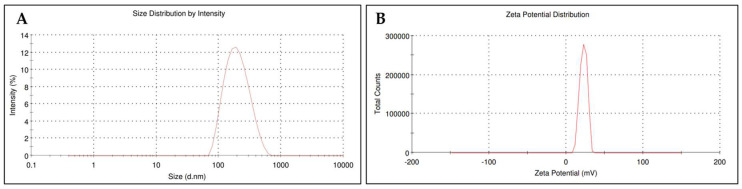
The optimized liposome F-07 particle size distribution profile (**A**) and Zeta potential peak (**B**).

The vesicular and zeta potentials of the liposome formulations increased with increasing cholesterol and DOTAP concentrations, as shown in Table 1. The vesicular size and zeta potential of the optimized F-07 formulation were found to be 190.3 ± 3.3 nm and 16.56 ± 2.51 mV, as depicted in Figure 4A and Figure 4B, respectively.

### 2.5. Microscopic Analysis by SEM and HR-TEM

**Figure 5:**
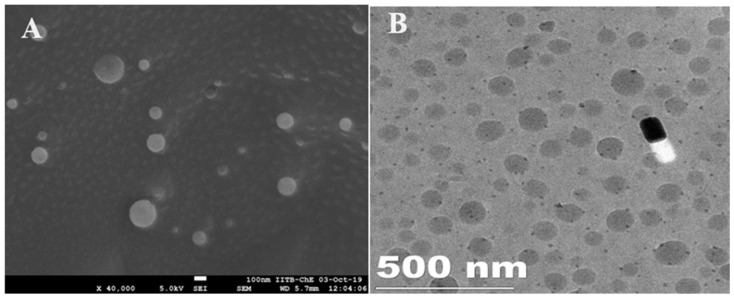
Scanning electron microscopy image (**A**) and HR-transmission electron microscopy image (**B**) of the optimized liposome formulation F-07.

The optimized liposomes encapsulating SIM were subjected to morphological examination using transmission electron microscopy (TEM) and scanning electron microscopy (SEM), as depicted in Figure 5A and Figure 5B, respectively. The micrographs revealed predominantly round or oval shapes with a narrow size distribution, and the liposomes displayed a non-aggregated state. The particle sizes observed in the TEM images were consistent with the measurements obtained through dynamic light scattering (DLS) using a zeta-sizer. Additionally, the three-dimensional SEM image of the optimized SIM-loaded liposomes confirmed their surface morphology, revealing nearly spherical structures with smooth surfaces. These observations confirm the successful development of a spherical nanosystem characterized by a size distribution in the nanosize range (<200 nm), indicating its potential suitability for topical delivery.

### 2.6. FTIR

**Figure 6:**
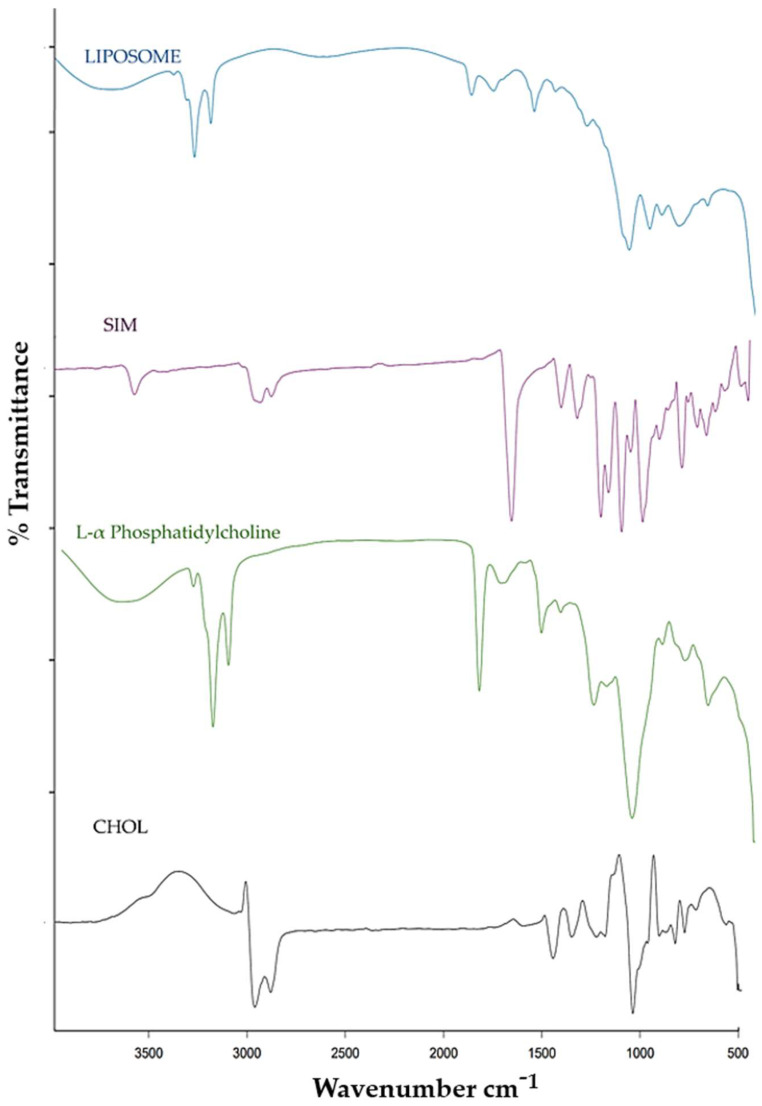
FT-IR overlay peaks of cholesterol, L-phosphatidylcholine, SIM, and optimized liposomes.

FT-IR spectroscopy was utilized to analyze the chemical structure of the SIM–liposome excipients and the pure drug, as shown in Figure 6. The spectra of cholesterol exhibited peaks at 1394.62 cm^−1^ (C-O stretching-alcohol functional group), 1596.45 cm^−1^ (alkene C=C stretching functional group), 2899.23 cm^−1^ (alkane C-H stretching functional group), and 3513.32 cm^−1^ (O-H stretching functional group). L-α phosphatidylcholine displayed characteristic peaks at 2922 cm^−1^ and 2822 cm^−1^ (CH2 and CH3 stretching in the fatty acid functional groups), 1733 cm^−1^ (C=O stretching as part of the ester functional group), and 1260 cm^−1^, 1100 cm^−1^, and 1034 cm^−1^ (P=O stretching functional group). Similar peaks were detected for SIM, including at 3549.62 cm^−1^ (O-H functional group), 2924 cm^−1^ (methylene C-H asymmetric and symmetric functional groups), 1701.27 cm^−1^ (ester C=O functional group), 1268.24 cm^−1^ (lactone -C-O-C functional group), 1165 cm^−1^ (ester -C-O-C- functional group), and 1072.46 cm^−1^ (secondary alcohol C-O functional group). The FTIR spectrum of the SIM-loaded liposomes exhibited all the identical peaks found for both the drug and the excipients.

### 2.7. XRD

**Figure 7:**
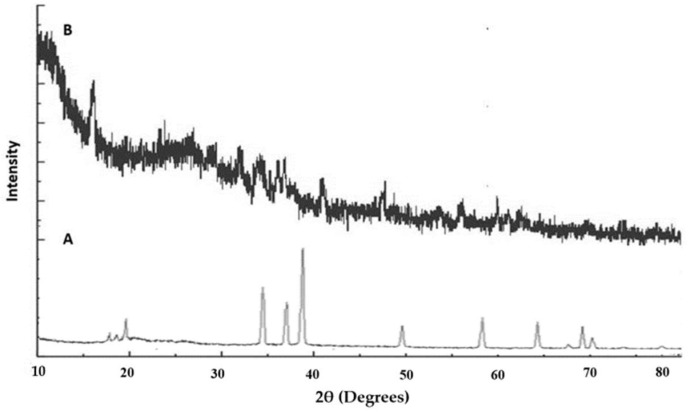
XRD overlay peaks of SIM (**A**) and F-07 optimized liposome gel (**B**).

The X-ray diffraction (XRD) patterns of the pure SIM and SIM-loaded liposomes are shown in Figure 7A,B. The XRD patterns of SIM show intense and evident diffraction peaks at diffraction angles (2θ) of 17.23°, 34.43°, 37.84°, 39.14°, 49.24°, 58.42°, 64.14°, 66.30°, 68.21°, and 71.23°, thus confirming its crystalline nature. All the characteristic peaks were absent in the liposomal formulation, ensuring that SIM was converted to an amorphous form with better solubility and encapsulation.

### 2.8. DSC

**Figure 8:**
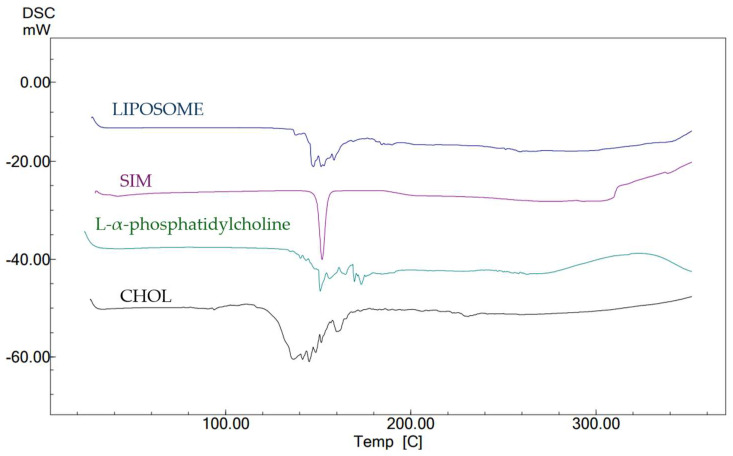
DSC thermograph overlay of cholesterol, L-α-phosphatidylcholine, SIM, and optimized liposomes.

The DSC thermograms showed a distinct peak at 132.09 °C, indicating the presence of cholesterol. The L-α phosphatidylcholine thermogram peaked at 180.32 °C. In comparison, the thermogram of SIM presented a distinct endothermic peak at 155.07 °C, indicating an increase in moisture content inside the sample. The thermogram of the optimized liposome formulation exhibited the anticipated endothermic peaks corresponding to the drug and polymer at their respective locations. Figure 8 demonstrates the absence of a substantial interaction between SIM and the polymers.

#### 2.8.1. Appearance, pH, and Spreadability

The optimized SIM liposomes incorporated into the gel exhibited a white color and opacity, and a pH of 6.3 ± 0.5 is considered suitable for topical preparations, aligning with the physiological pH of the skin and minimizing irritation upon application to the skin’s surface. The spreadability value of 4.2 ± 0.7 indicated efficient gel spreadability with minimal shear. This attribute is essential for patient compliance and for facilitating gel application to the skin.

#### 2.8.2. Viscosity

The rheological properties of topical gel formulations play a crucial role in delivering drugs onto or across the skin. These properties can significantly affect the spreadability, adhesion, diffusion of drugs, and permeation across the skin [21]. The rheological behavior of gels is essential for their mixing and flow characteristics, packaging, physical stability, and consumer acceptability. These properties of the gels can be influenced by factors such as temperature. In this study, the optimized SIM-loaded liposome gel was evaluated at 4 °C, 25 °C, and 40 °C, corresponding to dermal application and storage conditions.

**Figure 9:**
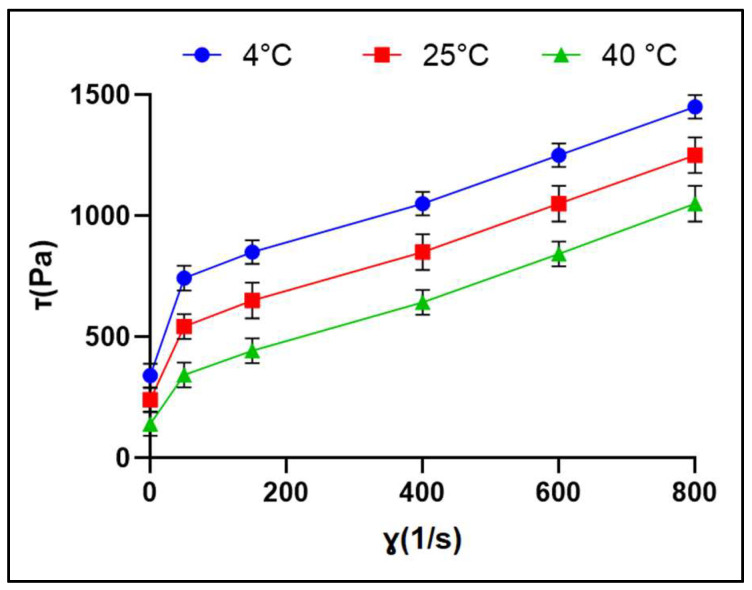
The graph displays the relationship between shear stress [τ] (measured in Pascals) and shear rate [ɣ] (measured in inverse seconds) for the optimized SIM liposome gel. Each value represents mean ± SD, and the experiment was performed in triplicate (*n* = 3).

The optimized SIM-loaded liposome gel demonstrated a pseudoplastic (shear-thinning) flow behavior, where the viscosity reduced as the shear rate increased, as illustrated in Figure 9. This behavior is advantageous for topical gel formulations, as it allows for easy spreadability and maximum coverage during application under shear stress. Under static conditions, the gel can return to its viscous form, providing good adhesiveness to the skin. The high viscosity of 278.4 Pa·s at a shear rate of 0.1 s^−1^ suggests that the developed gel has a high resistance to flow under low shear conditions, which is suitable for transdermal administration. These findings are consistent with previous studies on propranolol hydrochloride-loaded liposomal gels [22] and chitosome hydrogels [23], both of which exhibited pseudoplastic flow behavior with shear-thinning characteristics.

The pseudoplastic flow exhibited by the optimized SIM-loaded liposome gel is advantageous for various applications, particularly in the formulation of products where controlled flow properties are desirable. This behavior ensures the gel can maintain its structure and consistency during storage and application while also facilitating easy and uniform spreading on the skin surface. Hence, the rheological properties of the optimized SIM-loaded liposome gel, including its pseudoplastic flow behavior and high viscosity at low shear rates, are crucial for its performance and suitability as a topical formulation. These characteristics align with the desirable properties of topical gels, ensuring optimal spreadability, adhesiveness, and drug delivery on the skin [24].

#### 2.8.3. In Vitro Drug Release Study

**Figure 10:**
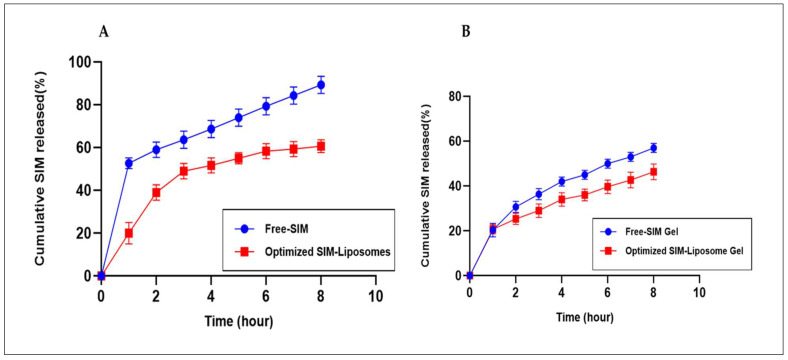
Drug release of free SIM and optimized SIM-loaded liposomes. (**A**) Release of free SIM incorporated into a gel and optimized SIM-loaded liposomes incorporated into a gel. (**B**). Each value represents mean ± SD, and the experiment was performed in triplicate (*n* = 3).

Figure 10A shows the release profiles of both free SIM and the optimized SIM-loaded liposomes. Significantly, the release kinetics of the free SIM were more rapid than those of the optimized SIM-loaded liposomes. Specifically, 54.32% of the drug was released after the first hour, increasing to 94.24% after eight hours for the free-SIM formulation. Meanwhile, the SIM–liposomal formulation exhibited a prolonged and sustained release pattern characterized by biphasic behavior. The swift initial release observed within the first two hours could be attributed to SIM being embedded within the fatty acid chains present in the bilayers of the liposome, facilitating rapid release when the liposomes disperse into the release medium; later, the drug release was controlled by diffusion within the swollen vesicles [25]. A gradual and sustained release followed, with only 57.05% of the drug being released after eight hours. This behavior aligns with the well-established role of liposomal systems as drug reservoirs, facilitating controlled and gradual drug release [25,26].

Figure 10B shows a comparison of the release profiles of the optimized SIM–liposome gel and free-SIM gel, which revealed that the drug release decreased in both cases. After 8 h, the released amounts were 58.02% and 48.59% for the free-SIM gel and optimized SIM–liposome gel, respectively. Similar findings have been documented for vesicular carriers incorporated in hydrogels, where the slow movement of the drug is due to the hindrance of drug diffusion facilitated by the polymeric structure of the hydrogel. This influences the release rate significantly and delays drug release compared to the free drug delivery system [27,28]. 

#### 2.8.4. In Vitro Drug Release Kinetic Modeling

The release data obtained from the study were analyzed using various kinetics models, including the zero-order, first-order, Higuchi, and Korsmeyer–Peppas models. The optimized liposome formulation demonstrated a release profile that aligned with the Higuchi model, indicating that the primary mechanism of drug release was diffusion from the carrier membrane, and the observed decrease in drug release rate is likely attributable to the influence of phosphatidylcholine, which increases the lipid layer’s thickness and modifies membrane fluidity [29]. Furthermore, the presence of cholesterol in the liposomes contributes to a reduction in membrane permeability, potentially contributing to the decelerated drug release kinetics [30]. In contrast, both the optimized SIM–liposome gel and the free-SIM gel followed the Korsmeyer–Peppas kinetic model. The calculated release index (n) for the SIM–liposome gel and free-SIM gel was approximately 0.1, falling below the model’s threshold of 0.5. This suggests a quasi-Fickian diffusion mechanism for drug release, implying that drug distribution occurred through the pores of the gel framework [31,32]. Table 2 displays the findings from the kinetic release investigation. 

### 2.9. Assessment of In Vivo Wound Healing Efficacy

#### 2.9.1. Evaluation of Wound Size

**Figure 11:**
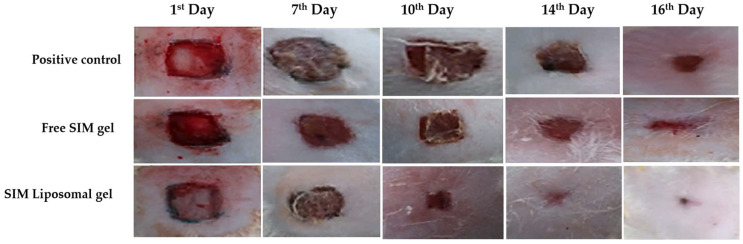
Wound healing progression was assessed at various time points, specifically on days 1, 7, 10, 14, and 16 post-wounding, across the different experimental groups.

**Figure 12:**
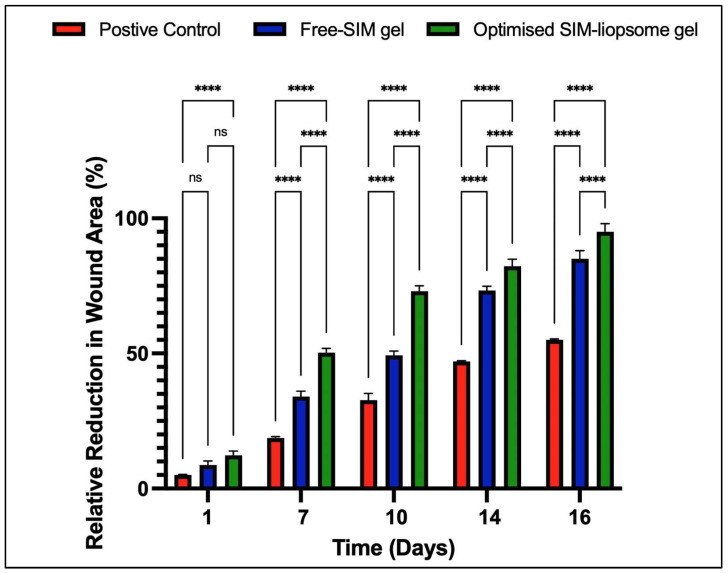
Advancement of wound healing in rats was examined within the various experimental groups at specified intervals of 1, 7, 10, 14, and 16 days following the initiation of the wound, with a sample size of 8 subjects in each group. Relative reduction in wound area of free-SIM gel and optimized SIM–liposomes gel was compared to positive control (data expressed as mean ± SD, *n* = 3; ns—non-significant, **** *p* < 0.0001).

The representation in Figure 11 illustrates that the wound size was reduced relative to that in the other groups. At the same time, Figure 12 provides a comprehensive depiction of the wound healing progression for various treatments across all groups from day 0 to day 16. The observed sequence of wound healing efficacy was as follows: positive control < free-SIM gel < optimized liposome gel. Specifically, the positive control exhibited a modest 59.67% reduction in wound area sixteen days post-wounding. In contrast, the optimized liposome gel demonstrated notable effectiveness in wound healing, resulting in a substantial relative decrease in wound area of 97% (*p* < 0.0001). Remarkably, compared to the free-SIM gel, the optimized liposome gel significantly reduced the wound size (*p* < 0.0001).

#### 2.9.2. Histopathology Study

**Figure 13:**
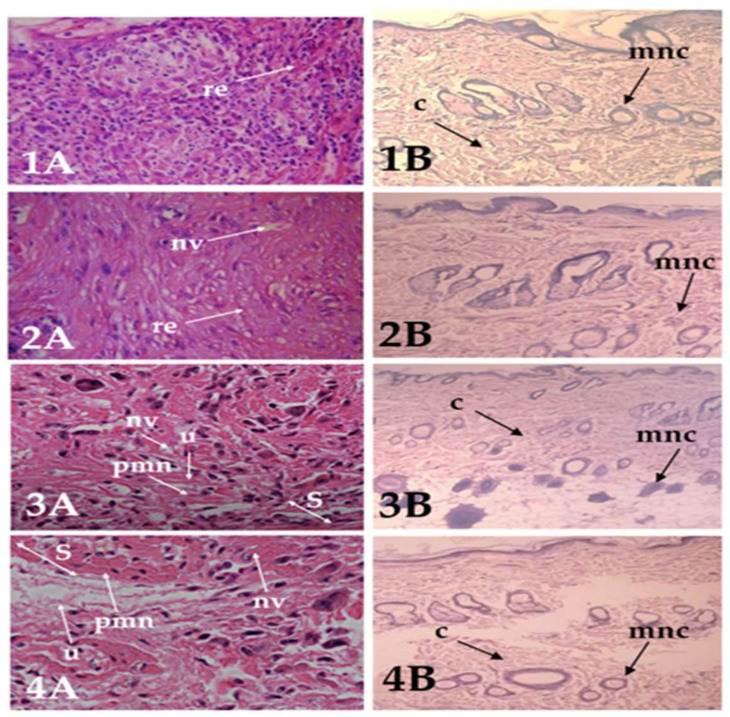
Histological perspective of wound healing and the restructuring of the epidermis and dermis in animals treated with the optimized liposome gel, free-SIM gel, positive control, or negative control. Skin slices display the epidermis and dermis stained with HE in image (**A**) and the dermis stained with VG in image (**B**), respectively. Group 1: 16-day-old wound tissue treated with optimized liposome gel. Group 2: 16-day-old wound tissue treated with pure drug incorporated into the gel. Group 3: 16-day-old wound tissue treated with USP povidone–iodine ointment 5% *w*/*w* (positive control). Group 4: negative control group, 16-day-old untreated group. The arrows represent many events in the process of wound healing: s, scar formation; re, re-epithelialization; c, collagen production; mncs, mononuclear cells; pmn, polymorphonuclear cells; and nv, neovascularization.

In this study, we demonstrated the efficacy of optimized SIM–liposome gel formulations in significantly improving wound healing outcomes compared to conventional treatments. Through wound histological analysis, we investigated how these formulations enhance the wound re-epithelialization process, showcasing their potential as advanced wound management strategies. The phenomenon of wound healing, encompassing the phases of inflammatory processes, cell proliferation, and remodeling, was observed and documented in the different experimental groups (Table 3). The histological findings can be confirmed by examining pictures of the tissues stained with hematoxylin and eosin (HE) and Verhoeff’s van Gieson (VG) (Figure 13).

The evaluation of wound healing processes among the experimental groups revealed varying responses. The optimized SIM–liposome gel and free-SIM gel groups exhibited moderate-to-severe effectiveness in scar formation, re-epithelialization, ulcer formation, collagen deposits, cell infiltration, and fibroblast proliferation. They also showed moderate neovascularization and polymorphonuclear cell presence across the healing phases. Previous studies support our findings: Ahmed et al. demonstrated re-epithelialization with SIM cubosomal nanoparticles [33]. Farghaly Aly et al. showed complete epithelialization with SIM polymeric nanoparticle gel [34], as well as minimal inflammation and mature collagen fiber formation. Varshosaz et al. detected thinning of the epithelium with an SIM gel, while SIM–micelle gel enhanced re-epithelialization, reduced inflammation, and promoted superior collagen content and skin tissue regeneration [12]. Kiani et al. revealed enhanced collagen deposition and skin rejuvenation with SIM-loaded liposomes in histological assessments of treated wounds [35]. Conversely, the positive and negative control groups displayed mild-to-moderate ulcer and scar formation, with absent-to-mild re-epithelialization and mild-to-moderate responses in collagen deposits, cell infiltration, fibroblast proliferation, and neovascularization. Notably, the positive control group demonstrated moderate remodeling, while the negative control group exhibited no remodeling. These findings emphasize the importance of formulation optimization in enhancing wound healing, particularly in promoting effective tissue remodeling and repair.

### 2.10. Stability Study

Ensuring the stability of liposomal formulations is crucial for their development. In this study, we investigated key parameters, including the mean particle size, zeta potential, and entrapment efficiency, over a 3-month period. The results from Table 4 indicate that the samples stored at 4 °C exhibited superior stability compared to those stored at 25 °C and 35 °C. However, the optimized formulation stored at 25 °C and 35 °C showed a slight increase in particle size, likely due to aggregation or swelling, a phenomenon supported by previous studies [36,37]. The zeta potential values decreased slightly during storage, attributed to the negative charge imparted by non-ionic surfactants like Span, which enhances electrostatic repulsion between liposomes, preventing aggregation [38]. Although there was a negligible reduction in entrapment efficiency, it may be linked to drug leakage from the vesicle surface [39]. Overall, no significant differences were observed in the mean particle size, zeta potential, and entrapment efficiency of the optimized liposomes stored at 4 °C, 25 °C, and 35 °C, suggesting optimal stability influenced partly by the carrier’s nature and constituents.

## 3. Conclusions

In the present study, SIM was successfully encapsulated within liposomal carriers using L-α-phosphatidylcholine, cholesterol, DOTAP, and Span 80. The Box–Behnken design–response surface methodology optimized the SIM-loaded liposomes. Polynomial models were obtained to predict the zeta potential, particle size, and encapsulation efficiency. The effects of these three variables were observed, i.e., increasing the concentration of DOTAP (X_1_) and cholesterol (X_3_) increased the zeta potential and particle size of liposomes, respectively, and increasing the concentration of Span 80 (X_2_) above 2.5% decreased the entrapment efficacy. The optimized formulation F-07 showed a zeta potential of 16.56 ± 2.51 mV, an entrapment efficiency of 95.6 ± 4.2%, and a particle size of 190.3 ± 3.3 nm. The drug release pattern of the optimized liposomes exhibited a biphasic nature, with an initial burst of release followed by sustained release. A reduced drug release rate was noted upon incorporating the formulations into the gel. These results suggest that the liposomes produced in this study have the potential to serve as carriers, providing both an initial dosage and prolonged release for therapeutic purposes. The optimized F-07 liposomes showed acceptable stability and superiority. In vivo investigations conducted on rat models revealed a substantial reduction in wound size, indicating the efficacy of the intervention in wound management. These results suggest the potential of the developed liposomal carriers as promising vehicles for delivering therapeutic agents in wound management.

## 4. Materials and Methods

### 4.1. Materials

#### 4.1.1. Chemicals

SIM was procured from Biocon Pvt. Ltd., Bangalore, India. L-α phosphatidylcholine (LPC), 1,2-dioleoyloxy-3-trimethylammoniumpropanchloride (DOTAP), Span 80, and cholesterol (COL) were obtained from Sigma Aldrich, Mumbai, India. Chloroform and methanol were procured from Merck, Mumbai, India.

#### 4.1.2. Animals

The experimental procedures involving animals adhered strictly to ethical guidelines and were conducted with approval (approval no. JSSCPM/CPT/IAEC/213/2017 dated 11 March 2017) from the Institutional Animal Ethics Committee (IAEC) at the JSS College of Pharmacy, Jagadguru Sri Shivarathreeshwara University (JSS University), Mysuru. It is a crucial process to calculate the number of animals before beginning a preclinical study, and it is a very important process to demonstrate its validity, accuracy, and reliability. The calculation of the number of animals before beginning the study is desired to test the intended research objective. We adopted the Precision Analysis Method for calculating the number of animals. Consequently, Albino Wistar rats of both genders, weighing between 200 and 250 g, were procured from Aditya Biosys, Bangalore, and subjected to protocols following the National Institutes of Health Guide for the Care and Use of Laboratory Animals. The animals received humane treatment throughout the study, including a routine pellet diet and unrestricted access to water.

### 4.2. Design of the Experiment

A Box-Behnken experimental design was used to evaluate the impact of excipients on the physicochemical characteristics of the liposomes. Specifically, the zeta potential, entrapment efficacy, and particle size were assessed as dependent parameters (Y) (Table 5). Three independent variables (X) were chosen: 1,2-dioleoyloxy-3-trimethylammoniumpropanchloride (DOTAP), Span 80, and cholesterol at three different levels, i.e., low, medium, and high (Table 5), along with 3 center points. A total of 15 randomized trial runs (Table 6) were performed for the current optimization research utilizing Design-Expert v.13.0 software (Stat-Ease Inc., Minneapolis, MN, USA) and response surface methodology (RSM) calculations. A linear model with quadratic terms was used to design all the response variables. Linear plots and 3D graphs were generated utilizing Design-Expert software. The significance of these effects on the variables was determined using an analysis of variance (ANOVA) [40,41]. The desired response criteria were set at ≥85% for entrapment efficiency, a particle size ≤200 nm, and a zeta potential ≥10 mV.

### 4.3. SIM-Encapsulated Liposome Preparation

**Figure 14:**
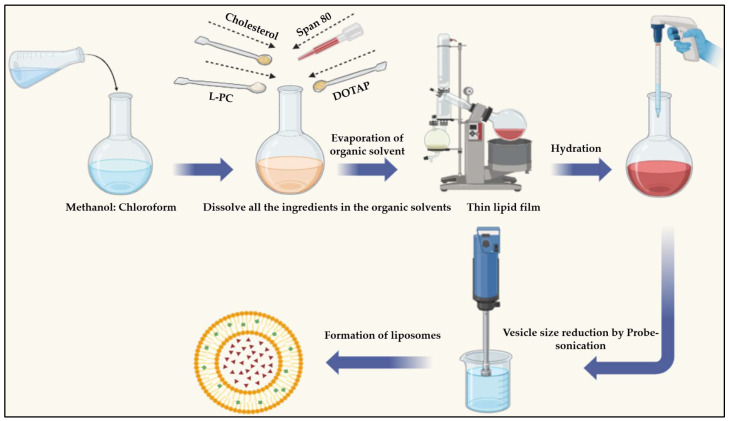
Schematic representation of the SIM-encapsulated liposome preparation.

The film hydration method was employed for the preparation of SIM-encapsulated liposomes, and the optimized factors provided in Table 3 were employed to develop drug-loaded liposomes. The detailed procedure is as follows: in a round-bottom flask, a weighed amount of the lipid component (L-phosphatidylcholine), cholesterol, DOTAP, Span 80, and SIM were dissolved in different chloroform and methanol combination ratios. A thin, dry lipid layer was developed on the interior surface of the flask through the gradual evaporation of organic solvents using a rotary evaporator (REMI, Mumbai, India) operating at 45 °C, a temperature exceeding the gel–liquid crystal transition temperature (Tc) of phospholipids. Subsequently, the dried lipid film was gradually hydrated in a rotary evaporator for three hours by adding 20 mL of phosphate-buffered saline (PBS) at a pH of 7.4, ultimately leading to the formation of a liposome [42]. The mixture was probe-sonicated (Sonic Vibra Cell, Newtown, CT, USA) for 10 min at a 40% amplitude to achieve nanosized liposomes and minimize their size. A schematic representation of the SIM-encapsulated liposome formulation is provided in Figure 14 for a better understanding. 

### 4.4. Analytical Characterization of the Liposome-Encapsulated SIM Formulation

#### 4.4.1. Determination of Entrapment Efficiency

The procedure involved centrifuging hydrated vesicular dispersions and any unencapsulated SIM (SIM) using a cooling centrifuge (Z216-MK, Hermile, Benchmark, Lodi, NJ, USA) at 4 °C and 15,000 rpm for 30 min. Following centrifugation, the resulting supernatant was diluted with phosphate buffer (pH 7.4) and subsequently analyzed for its SIM content using a spectrophotometer (UV spectrophotometer, 1800/PC, Shimadzu, Kyoto, Japan) at a specific wavelength of 237 nm [43]. The encapsulation efficiency (EE%) was determined using the following equation, which involved deducting the quantity of unencapsulated SIM from the initial total amount of the drug that had been incorporated.

$$\mathrm{Entrapment}\;\mathrm{efficiency}\;\left(\%\right)=\frac{\mathrm{Amount}\;\mathrm{of}\;\mathrm{drug}\;\mathrm{entrapped}}{\mathrm{Total}\;\mathrm{amount}\;\mathrm{of}\;\mathrm{drug}}\times 100$$

#### 4.4.2. Evaluation of Vesicle Size and Zeta Potential

The dynamic light scattering (DLS) method (Zetasizer, Nano ZS90, Malvern, UK) was utilized to assess the vesicle size and zeta potential (ZP) of the formulations. The samples were adequately diluted with distilled water and measured at 25 °C. To achieve precise analysis, the formulations were diluted 20 times with deionized water and evaluated at an angle of scattering of 90° at room temperature. Similarly, for zeta potential (ZP) determination, identical samples were introduced into the electrophoretic cell before analysis commenced. Each analysis was conducted in triplicate, and the results were recorded as the mean values along with their standard deviations (mean ± SDs).

#### 4.4.3. Scanning Electron Microscopy (SEM)

The morphology of the SIM-loaded liposomes was examined using SEM (Zeiss EVO LS 15, Smart SEM 5.05, Oberkochen, Germany). The specimens were positioned on an aluminum mount and subjected to gold coating using a high-vacuum evaporator in an argon atmosphere, and SEM analysis was subsequently performed [44,45,46].

#### 4.4.4. High-Resolution Transmission Electron Microscopy (HR-TEM) Analysis

HR-TEM (JEOL, JM 2100, Tokyo, Japan) was employed to investigate the surface morphology of liposomes encapsulating SIM. A copper grid was used to hold a small quantity of the liposomal suspension, which was then dried for approximately 3 min before being introduced into the microscope for imaging purposes.

### 4.5. FTIR, XRD, and DSC

The KBR pellet method was used to analyze the interactions of the pure drugs, excipients, and liposomal formulations with the excipients using an FTIR spectrophotometer (FTIR-8400s, Shimadzu, Kyoto, Japan). XRD (Rigaku, Tokyo, Japan). This instrument was used to assess the drugs, excipients, and SIM-loaded liposomes within a 2θ range of 2–45°, utilizing a current of –35 mA and a voltage of −40 kV. DSC (DSC-60, Shimadzu, Kyoto, Japan) was used for the thermal studies. SIM-loaded liposomes (10 mg) were introduced into the specimen holder and analyzed at a heating rate of 20 °C min^−1^ over a temperature range of 20 °C to 400 °C [47,48].

### 4.6. Incorporation of Liposomes into the Gel

The achievement of topical drug delivery for a liposomal formulation necessitates the incorporation of an appropriate gelling agent to ensure favorable rheological and bioadhesive properties [49]. To prepare the SIM-loaded liposomal gel, the optimized liposomes were integrated into a Carbopol-940-based hydrogel matrix. The Carbopol gel base was formulated by dispersing 1 g of Carbopol-940 (Loba Chemie, Mumbai, India) in 100 mL of PBS (pH 7.4) at a concentration of 1% *w*/*v*. After complete dispersion, the mixture was left at room temperature for 24 h. Triethanolamine (0.5% *w*/*w*) was then added drop by drop to adjust the basicity of the formulation [50]. Subsequently, with continuous stirring, the liposomal dispersion and gel base were combined in a 1:1 ratio. The resulting gel was left at room temperature for 2 h before further processing [51].

### 4.7. Evaluation of the Optimized SIM-Loaded Liposome Gel

#### 4.7.1. Appearance

The visual characteristics of the optimized liposomal gel loaded with SIM were evaluated through visualization. Gel samples, ranging from 1 to 2 g, were carefully placed on a watch glass. Subsequent visual inspections were conducted following storage at controlled temperatures of 25 ± 2 °C, 4 ± 2 °C, and 40 ± 2 °C. These assessments were carried out at specific intervals, including 1 day and 1, 2, and 3 months, to discern any notable changes in the gel’s appearance during the prescribed timeframes.

#### 4.7.2. Determination of pH

The pH of the optimized liposomal gel containing SIM was evaluated utilizing a digital pH meter (Mettler Toledo MP 220, Greifensee, Switzerland) at a consistent temperature of 25 °C. One gram of the gel was suitably diluted and dispersed in 10 mL of distilled water to conduct the measurement. The pH values were subsequently measured in triplicate, and the average reading for each formulation was calculated.

#### 4.7.3. Spreadability

To evaluate the spreadability of the SIM-containing optimized liposomal gel, 0.5 g of the gel was spread over the center of a 5 cm diameter watch glass. A second watch glass was carefully placed over the first to ensure even spreading, and the assembly was left undisturbed for five minutes. The spreadability of the resultant spread circles was then quantified by measuring their diameters [52].

#### 4.7.4. Viscosity

Viscosity assessments were conducted using a stress rheometer (Anton-Paar GmbH, Graz, Austria) and RheoCompass^TM^ version 1.33.491 (Anton-Paar GmbH, Graz, Austria) software. This was carried out under three distinct temperature conditions: 4 ± 0.1 °C, 25 ± 0.1 °C, and 40 ± 0.1 °C. Before analysis, the optimized SIM liposome gel samples underwent a 300 s equilibration period. The plate rotor was meticulously adjusted to maintain the space between the rotor plate and the bottom plate at 1.00 mm. A curve that plots the relationship between shear stress and shear rate, ranging from 0.1 to 1000 s^−1^, was developed to determine the dynamic viscosities of the samples [53,54].

#### 4.7.5. Drug Release Study

The in vitro release profiles of SIM from several formulations, including free SIM, optimized SIM-loaded liposomes, SIM incorporated into gel, and optimized SIM–liposome gel, were studied using Franz diffusion cells with a receptor volume of 25 mL. Each sample containing 2 mg of SIM was individually deposited on a dialysis membrane (D9777 Dialysis Tubing Cellulose Membrane, Merk, Mumbai, India, with a molecular weight cut-off of 12,000–14,000) in four different diffusion cells. The dialysis membrane was located between the receptor and donor chambers [55]. To maintain sink conditions, a phosphate buffer solution with a pH of 7.4 containing 0.05% sodium dodecyl sulfate was utilized as the release medium. The release study was conducted using a Franz cell positioned between a magnetic stirrer operating at 160 rpm and a water bath set at 37 ± 1 °C. [39]. Samples were retrieved from the receptor compartment at defined time intervals (0, 1, 2, 3, 4, 5, 6, 7, and 8 h), with 1 mL of sample taken each time and immediately replaced with an equal volume of fresh buffer at the same temperature to maintain a constant volume. The collected samples were analyzed using a validated UV spectrophotometric method (UV spectrophotometer, 1800/PC, Shimadzu, Japan) at 237 nm, with the buffer serving as a blank. The cumulative release of the drug was calculated and plotted against time [39,55]. All measurements were taken in triplicate and represented as mean ± SD.

#### 4.7.6. Drug Release Kinetics

Several kinetic models were used to analyze the drug release data to determine the order and mechanism controlling drug release from the system. The data were fitted to the zero-order, first-order, Higuchi model, and Korsmeyer–Peppas models using the DD solver.xla add-in for Microsoft Excel 2021 version 2404 [56].

### 4.8. Assessment of the In Vivo Wound Recovery Process

#### 4.8.1. Experimental Design

The wound-healing efficacy of the SIM-loaded liposome carrier was assessed using a full-thickness wound model. Four groups comprising male and female albino Wistar rats (*n* = 8) were established for the study. Group I was the negative control and consisted of healthy and uninjured animals. Groups II–IV underwent wound excision and anesthesia using ketamine + xylazine at a dose of 100 mg/kg (K) + 10 mg/kg (X) intraperitoneally. If animals exhibited responsiveness to touch, redosing occurred with 30% of the initial ketamine dose alone, without additional xylazine. Full-thickness wounds were made on the clipped dorsal skin of the rats using a sterilized biopsy punch needle (Ribbel International Ltd., Haryana, India) to make circular incisions with a diameter of 5 mm. Animals in Groups II, III, and IV received a once-daily application of standard 5% *w*/*w* povidone–iodine ointment USP (positive control), SIM solution mixed with gel (pure drug), or optimized SIM–liposome dispersion combined with gel (test formulation), respectively, for a total treatment duration of 16 days. The SIM concentrations of all the prepared gels were adjusted to 1%.

#### 4.8.2. Evaluation of Wound Size

The contraction of the wound serves as a crucial indicator of the healing process. To assess wound healing progress, images of the wound areas were captured with a smartphone camera (Redmi Note 11T, 15-megapixel resolution), keeping the image capturing parameters constant (like subject distance, focusing area, capturing resolution, etc.). The captured images were transferred to a computer equipped with ImageJ software v. 1.54i (National Institutes of Health, USA, http://rsbweb.nih.gov/ij/download.html accessed on 23 September 2023), and wound areas were measured manually using an ImageJ plugin on days 1, 7, 10, 14, and 16 post-injury [57]. Quantification of wound recovery involved calculating the percentage decrease in wound area over the 16-day experimental period using a specific equation:

$$\mathrm{Relative}\;\mathrm{reduction}\;\mathrm{in}\;\mathrm{wound}\;\mathrm{area}\;\left(\%\right)=\frac{\left(\mathrm{Ao}-\mathrm{At}\right)}{\mathrm{Ao}}\times 100$$

where the wound size at time zero (Ao) and time t are represented.

#### 4.8.3. Histopathology

Each biopsy specimen from the experimental groups underwent a standardized protocol, starting with rinsing in distilled water. Subsequently, the specimen was dehydrated using a series of alcohol dilutions—methanol, ethanol, and absolute ethanol. Following dehydration, the specimens were clarified with xylene and embedded in paraffin in a hot-air oven set at 56 °C for 24 h. The resulting paraffin–beeswax tissue blocks were precisely sectioned into 5 μm thick slices using a microtome. These sections were delicately mounted on glass slides, subjected to deparaffinization, and stained with hematoxylin and eosin (HE) and van Gieson (VG). The stained sections were then examined under a Leica Qwin 500 Image Analyzer, which includes a Leica DM-LB microscope equipped with a JVC color video camera connected to a computer system (Leica Q 500IW). The sections were graded as mild (+), moderate (++), or severe (+++) for epidermal or dermal remodeling. The evaluation of epidermal or dermal remodeling included an assessment of fibroblast migration, mononuclear and polymorphonuclear cells, neovascularization, and collagen production within the epidermis. Van Gieson-stained sections were specifically analyzed to evaluate collagen production. Subsequently, the results were compiled and categorized based on the stages of wound healing—inflammation, proliferation, and remodeling—for all experimental groups. The histological analysis was conducted in a blinded manner to ensure an impartial assessment of the wounded tissue samples across all experimental groups [58].

### 4.9. Stability Studies

Optimized SIM-loaded liposomes were utilized for storage stability studies. Using glass vials, a 10 mL sample of the SIM–liposome dispersion with a 2 mg/mL drug concentration was kept at 4 °C and 25 °C for three months. With a sampling frequency of once per month, the stability test was assessed based on the particle size, zeta potential, and EE% found in the dispersion [59].

### 4.10. Statistical Analysis

GraphPad Prism 8.0.2 software (GraphPad Software Inc., San Diego, CA, USA) was used to statistically analyze all the recorded research data. For independent experiments, the results are reported as the mean ± SD (*n* = 3 to 15). One-way ANOVA with post hoc analysis employing Dunnett’s Multiple-Comparisons Test was used to assess differences amongst the groups. *p* < 0.05 was considered a statistically significant difference among all the groups [ns—non-significant, * *p* < 0.05, ** *p* < 0.01, *** *p* < 0.001, and **** *p* < 0.0001 (in comparison to the control group)].

## Figures and Tables

**Table 1:** Optimal levels of independent variables: predicted vs. experimental outcomes.

Independent Variables	Optimized Level	Dependent Variables	Predicted Values	Experimental Values
**DOTAP (mg)**	7.36%	%EE	93.25	95.6 ± 4.2
**Span 80 (%*w*/*w*)**	2.36	Particle size (nm)	187.75	190.3 ± 3.3
**Cholesterol (mg)**	99.78	Zeta potential (mV)	15.86	16.56 ± 2.5

**Table 2:** Release kinetic models of free SIM incorporated into gel, optimized SIM-loaded liposomes, and optimized SIM-loaded liposomes incorporated into gel.

Kinetic Model					
Formulation	Parameters	Zero-Order	First-Order	Higuchi	Korsmeyer-Peppas
Free-SIM gel	R^2^	0.7616	0.7756	0.9254	0.9727
	K	0.2331	0.0331	2.4072	16.5433
	n	−	−	−	0.1732
Optimized SIM-loaded liposomes	R^2^	0.1670	0.9341	0.9961	0.9781
	K	0.1467	0.0013	1.6091	6.3751
	n	−	−	−	0.2561
Optimized SIM–liposome gel	R^2^	0.7241	0.7408	0.9152	0.9536
	K	0.04828	0.0005	0.4351	6.2619
	n	−	−	−	0.1641

n represents the diffusion or release exponent, which is specifically used in the Korsmeyer–Peppas model. k = release constant.

**Table 3:** Histopathological assessment of wound sections revealed the effects of various formulations on the phases and process of wound healing.

Groups		Wound Healing Process							Healing Process	
	S	RE	U	C	MNC	NV	PMN	I	P	R
Optimized Liposome-gel	++	++	++/+++	++	+/+++	++/+++	++/+++	++/+++	+++	++/+++
SIM-gel free	++	++	++/+++	++	++/+++	+/++	++/+++	++	++	−/+
Positive control	++	−	++	++	+/++	++	+/++	++	++	−
Negative control	+	−	++	+	+/++	+	+	+	+	−

S, scar; RE, re-epithelialization; U, ulcus; C, collagen deposit; MNC, mononuclear cells; NV, neovascularisation; PMV, polymorphonuclear cells; I, inflammatory phase; P, proliferative phase; and R, remodeling phase. The sections stained with HE and VG were assessed for epidermis and dermis remodeling and scored as very mild (−), (mild (+), moderate (++), or severe (+++).

**Table 4:** Storage stability studies of optimized SIM-encapsulated liposome formulation.

Time (Days)	Storage Temperature								
	4 °C			25 °C			35 °C		
	Particle Size (nm) *	Zeta Potential (mV) *	Entrapment Efficiency (%) *	Particle Size (nm) *	Zeta Potential (mV) *	Entrapment Efficiency (%) *	Particle Size (nm) *	Zeta Potential (mV) *	Entrapment Efficiency (%) *
0	190.3 ± 5.3	+16.56 ± 2.2	95.6 ± 4.2	183.3 ± 7.3	+18.32 ± 4.2	93.4 ± 6.2	194.4 ± 6.3	+17.71 ± 4.1	94.7 ± 5.6
15	195.6 ± 3.1	+14.72 ± 4.7	93.2 ± 6.7	186.9 ± 4.1	+16.67 ± 8.3	91.3 ± 8.4	204.3 ± 4.2	+15.41 ± 3.6	92.4 ± 7.9
30	194.7 ± 8.3	+15.33 ± 9.4	91.4 ± 3.5	188.4 ± 6.2	+14.21 ± 7.4	88.3 ± 7.2	205.4 ± 8.4	+14.64 ± 8.1	89.2 ± 3.2
60	196.9 ± 4.7	+13.52 ± 3.7	90.7 ± 5.1	190.21 ± 3.1	+11.42 ± 9.2	86.4 ± 9.4	201.4 ± 6.7	+11.45 ± 7.3	86.7 ± 8.6
90	193.2 ± 6.2	+12.81 ± 7.5	89.4 ± 6.8	195 ± 7.5	+8.84 ± 3.2	85.3 ± 4.2	212.4 ± 3.2	+7.71 ± 9.4	85.4 ± 4.7

* Each value represents mean ± SD, and the experiment was performed in triplicate (*n* = 3).

**Table 5:** Box-Behnken design with dependent and independent variables.

Dependent Variables with Their Limitations, along with Independent Variables with Their Level		
Variables	Level	
	Low [−1]	High [+1]
**Independent variables**		
DOTAP (mg)	2.5	7.5
Span 80 (%)	2.5	7.5
Cholesterol (mg)	80	120
**Dependent variables**		
Zeta Potential [mV]	Maximize	
Entrapment Efficacy [%]	Maximize	
Particle size [nm]	Minimize	

**Table 6:** Box-Behnken design with results.

Runs	Factor 1 A: DOTAP mg	Factor 2 B: Span 80 %*w*/*w*	Factor 3 C: Cholesterol mg	RESPONSE 1 Zeta Potential mV	Response 2 Entrapment Efficacy (%)	Response 3 Particle Size nm
F-1	2.5	5	80	−1.45 ± 1.53	87.09 ± 2.21	170.2 ± 3.62
F-2	5	7.5	80	4.22 ± 1.82	83.67 ± 3.74	150.5 ± 5.55
F-3	5	7.5	120	3.56 ± 1.46	80.32 ± 4.95	350.2 ± 5.47
F-4	5	2.5	120	4.5 ± 1.73	96.32 ± 2.43	370.4 ± 6.89
F-5	5	2.5	80	5.26 ± 1.87	94.93 ± 3.77	160.7 ± 3.11
F-6	5	5	100	7.32 ± 1.92	86.34 ± 3.82	245.6 ± 2.32
F-7	7.5	2.5	100	16.56 ± 2.51	95.6 ± 4.21	190.3 ± 3.34
F-8	5	5	100	6.54 ± 1.55	88.34 ± 2.16	245.5 ± 4.26
F-9	7.5	5	80	14.23 ± 2.72	86.54 ± 3.45	140.9 ± 3.53
F-10	2.5	7.5	100	−3.62 ± 1.91	80.34 ± 2.51	280.3 ± 6.72
F-11	7.5	5	120	13.24 ± 2.25	87.34 ± 3.35	376.3 ± 7.81
F-12	5	5	100	2.245 ± 1.86	89.5 ± 4.72	270 ± 4.21
F-13	2.5	5	120	−4.342 ± 1.47	87.65 ± 3.96	360.5 ± 7.14
F-14	2.5	2.5	100	−2.123 ± 1.23	95.43 ± 5.28	235.4 ± 3.52
F-15	7.5	7.5	100	12.23 ± 2.57	81.23 ± 2.51	244.7 ± 4.85

## Data Availability

All the data supporting the conclusions of this research are contained within the article itself.
